# The Effect of Agglomeration on Arsenic Adsorption Using Iron Oxide Nanoparticles

**DOI:** 10.3390/nano12091598

**Published:** 2022-05-09

**Authors:** William R. Diephuis, Anna L. Molloy, Lindsey L. Boltz, Tristan B. Porter, Anthony Aragon Orozco, Reina Duron, Destiny Crespo, Luke J. George, Andrew D. Reiffer, Gabriela Escalera, Arash Bohloul, Carolina Avendano, Vicki L. Colvin, Natalia I. Gonzalez-Pech

**Affiliations:** 1 Department of Chemistry, Hope College, Holland, MI 49423, USA; william.diephuis@hope.edu (W.R.D.); anna.molloy@hope.edu (A.L.M.); lindsey.boltz@hope.edu (L.L.B.); tristan.porter@hope.edu (T.B.P.); anthonyaragon03@gmail.com (A.A.O.); redu062@hps21.org (R.D.); destinycrespo05@gmail.com (D.C.); luke.george@hope.edu (L.J.G.); andrew.reiffer@hope.edu (A.D.R.); 2 Department of Chemistry, Rice University, Houston, TX 77005, USA; gescalera10@gmail.com (G.E.); arashbohloul@gmail.com (A.B.); 3 Children’s Environmental Health Initiative, University of Notre Dame, South Bend, IN 46556, USA; cavendan@nd.edu; 4 Departments of Chemistry and Engineering, Brown University, Providence, RI 02912, USA; vicki_colvin@brown.edu

**Keywords:** iron oxide nanoparticles, agglomeration, arsenic remediation, adsorption, column experiments, clusters of nanoparticles

## Abstract

The presence of arsenic in groundwater and other drinking water sources presents a notable public health concern. Although the utilization of iron oxide nanomaterials as arsenic adsorbents has shown promising results in batch experiments, few have succeeded in using nanomaterials in filter setups. In this study, the performance of nanomaterials, supported on sand, was first compared for arsenic adsorption by conducting continuous flow experiments. Iron oxide nanoparticles (IONPs) were prepared with different synthetic methodologies to control the degree of agglomeration. IONPs were prepared by thermal decomposition or coprecipitation and compared with commercially available IONPs. Electron microscopy was used to characterize the degree of agglomeration of the pristine materials after deposition onto the sand. The column experiments showed that IONPs that presented less agglomeration and were well dispersed over the sand had a tendency to be released during water treatment. To overcome this implementation challenge, we proposed the use of clusters of iron oxide nanoparticles (cIONPs), synthesized by a solvothermal methodology, which was explored. An isotherm experiment was also conducted to determine the arsenic adsorption capacities of the iron oxide nanomaterials. cIONPs showed higher adsorption capacities (121.4 mg/g) than the other IONPs (11.1, 6.6, and 0.6 mg/g for thermal decomposition, coprecipitation, and commercially available IONPs, respectively), without the implementation issues presented by IONPs. Our results show that the use of clusters of nanoparticles of other compositions opens up the possibilities for multiple water remediation applications.

## 1. Introduction

According to the World Health Organization (WHO), water containing arsenic concentrations above 10 µg/L should be avoided to preserve human health [1]. Long-term consumption of water with arsenic levels above this proposed level may lead to health concerns including cancer, heart disease, disruption of cell function, etc. [2]. While these health risks may seem negligible to countries with advanced water filtration processes, the WHO estimates that nearly 150 million people consume arsenic-contaminated water above the suggested level. While many techniques have been suggested for arsenic-contaminated water remediation, many of the proposed methods are not feasible for various reasons, including poor removal of As(III), inadequate disposal techniques, and cost efficiency [3,4,5].

In recent years, the utilization of metal oxide nanomaterials as heavy-metal adsorbents has shown better adsorption performance than bulk materials [6,7,8,9]. The enhanced properties of nanomaterials have also been utilized in other applications such as photocatalysis, environmental sensors, and adsorption of other pollutants [10,11,12]. The increased surface area and their magnetic properties have made iron oxide nanomaterials ideal for arsenic removal [13,14]. Iron oxide nanomaterials are very efficient at removing arsenic in batch adsorption experiments [15,16,17,18] due to the strong Fe-O-As bonds formed during adsorption [19]. However, very few studies have used IONPs in pilot studies with continuous flow experiments with real water conditions [20]. A significant study by Farrell et al. demonstrated successful pilot-scale adsorption of arsenic with commercial-grade iron-oxide nanoparticles (IONPs) [21]. Further, Gonzalez-Pech et al. compared the performance of commercial-grade IONPs to other known commercial arsenic sorbents under continuous flow experiments. This study found that IONPs are a feasible alternative to other sorbents, but are not cost-effective and produce high levels of waste when commercial-grade [22]. While these results are promising regarding the use of IONPs for arsenic remediation, there are two key drawbacks: (1) the ability to manipulate and modify the commercial nanomaterials and (2) their cost-effectiveness. The ability to manipulate and modify the nanomaterials directly correlates with the effectiveness of the iron-oxide materials in adsorbing arsenic. As previously discussed, two of the key aspects that determine the adsorption capacity of nanomaterials are the size and surface area [23,24]. In general, as nanoparticle size decreases and surface area increases, their adsorption capacity increases. Apart from the size of the individual nanoparticles, the way in which the particles agglomerate can also influence the functionality of the materials. When looking at commercially produced nanoparticles, they tend to agglomerate tightly, forming aggregates of particles around 100 µm. However, when producing nanomaterials in a laboratory setting, different parameters may be manipulated to regulate the agglomeration patterns of nanomaterials.

More recently, Molloy et al. conducted a study to better understand agglomeration regulation among laboratory-produced nanomaterials and were able to effectively control aggregation in samples [25]. In batch experiments, it was found that decreased agglomeration, and thus an increased usable surface area, resulted in more effective arsenic adsorption. However, once implemented in a column setting, the lack of agglomeration appeared to be maladaptive, as leaching occurred and prevented effective arsenic removal. While some research has demonstrated that the controlled agglomeration of nanoparticles can result in an increased arsenic removal efficiency [26], no study has focused on the effect that the agglomeration of nanomaterials has on continuous flow conditions. Therefore, in this study, we analyzed the effect of using nanoparticles with different grades of agglomeration on their arsenic removal behavior when deposited on a sand support media. Based on our observations, we implemented clusters of nanoparticles synthesized via a solvothermal method, with the goal of maximizing functional surface area while limiting column-related leaching as seen with the other nanomaterials.

## 2. Methods

### 2.1. Materials

#### 2.1.1. Synthesis and Characterization of IONPs

Detailed information for the synthesis and characterization of all the nanomaterials used can be found in the Supplementary Materials.

#### 2.1.2. Arsenic Adsorption Experiments

The arsenic stock solution was prepared by dissolving arsenic (V) oxide (As2O5, +99.9%) in DI water with 4 g/L sodium hydroxide (NaOH, 99.99% trace metals basis). Solutions for experiments were prepared by dilution of stock solutions with DI water; pH was adjusted to 7 with nitric acid (HNO3, 70%, ≥99.999% trace metals basis) and sodium hydroxide (NaOH, pellets, semiconductor grade, 99.99% trace metal basis). As the stock solution is prepared from arsenic (V) oxide and adjusted to a pH 7, it is expected that HAsO_4_^2−^ is the main species present in the solutions. All reagents were purchased from Sigma-Aldrich (Saint Louis, MO, USA).

#### 2.1.3. Column Experiments

Glass wool for laboratory use was purchased from Sigma-Aldrich (Saint Louis, MO, USA). Ottawa sand (Fisher chemical) was procured from Fisher Scientific (Waltham, MA, USA). Borosilicate glass columns measuring 10 mm × 100 mm and 10 mm × 400 mm (Omnifit Labware) and including the adjustable end piece assembly and 10 mm × 50 μm PTFE frits were purchased from Diba Industries Ltd. (Cambridge, UK). Masterflex L/S Computer-Compatible Digital pumps (with Easy-Load 3 Pump Head, 600 rpm) and Masterflex tubing (Tygon, LFL, L/S 14) were acquired from Cole-Palmer (Vernon Hills, IL, USA).

### 2.2. Rapid Small Scale Column Tests

The columns were packed as described below depending on the active material; Figure S1 shows a general description of all packed columns. Once packed, DI water was run in an ascendant flow overnight to wash out all the air bubbles. The flow was changed from ascending to descending immediately before starting to feed the column with the arsenic solution (100 ppb, pH 7). Once running, the flow rate was kept at an empty bed contact time (EBCT) of 2 min. The flow rate was monitored every hour by weighing the water flow per minute. When needed, the programmed flow rate in the pump was increased. The experiment was aborted if the programmed flow rate had to be increased more than three times the desired flow rate. Every determinate time, 1 bed volume of sample was collected. Then, it was filtered using a 0.45 μm PES syringe filter and acidified. Arsenic was measured using inductively coupled plasma–mass spectrometry (ICP–MS) or inductively coupled plasma–optical emission spectroscopy (ICP–OES).

#### 2.2.1. Packing Solid Nanoparticles

Solid nanoparticles were dispersed on sand as a support medium. Mixtures of 20 wt% were used for columns for most nanoparticles. After adding the nanoparticles to the support medium in a 15 mL scintillation vial, the tube was stirred vigorously by hand for 1 min, followed by 24 h of agitation in a mechanical mixer. Finally, the mixture was introduced in the column as active material in the previous section.

#### 2.2.2. Packing Nanoparticles Synthesized via Thermal Decomposition

As the thermal decomposition synthesis generates small amounts of nanomaterials, the active bed for this type of nanomaterial was kept at 1 wt%. Prior to packing, thermal decomposition IONPs were deposited onto the sand. After the excess hexanes evaporated, the active material was exposed to super hydride for coating removal, as described in Supplementary Materials Section 2.4. Figure S2 shows SEM images of the thermal decomposition IONPs deposited onto the sand surface after the treatment with super hydride. Following this, the active bed was introduced to the columns for flow experiments.

### 2.3. Batch Experiments for Arsenic

In general, the performance of each material was measured by the difference between the initial and final solutions. Arsenic values were measured with ICP–OES.

#### 2.3.1. Adsorption Isotherms Using Solids

A known amount of solid was weighed in a 50 mL centrifuge tube (Figure S3i); then, 40 mL of the interest solution was added. Mixing at room temperature was then carried out in a mechanical mixer for 4 h (Figure S3ii) unless a different duration is stated. Subsequently, 10 mL of the supernatant was filtered with a 0.45 μm PES syringe filter (Figure S3iii). Finally, the sample was acidified with HNO_3_ trace metals and analyzed using ICP–OES to determine the arsenic concentration (Figure S3iv). For a single isotherm curve, at least 5 points were needed; weights between 4 and 200 mg were selected and repeated in triplicate.

#### 2.3.2. Adsorption Isotherms Using IONP Solutions

For isotherms with nanoparticles prepared by thermal decomposition, the procedure was different. IONPs were suspended in hexanes, and the volume added was usually smaller than 0.1 mL (depending on the sample nanoparticle concentration) (Figure S4i). As Figure S4ii shows, during mixing, the color of the solution was homogeneous. At the end of the procedure, some color was retained in the aqueous phase, as shown in Figure S4iii. When possible, the organic phase was separated by a transfer pipette before filtration. After filtration, the solution was left open for 30 min to ensure that the hexanes evaporated before acidification.

## 3. Results and Discussion

### 3.1. Effect of Agglomeration of IONPs Deposited onto Sand in Column Experiments

**Figure 1:**
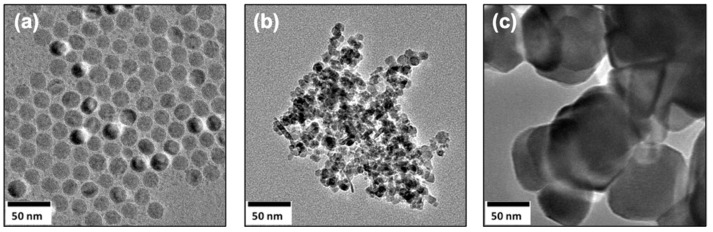
**IONPs prepared by different methods of synthesis:** TEM images of magnetite nanoparticles prepared by thermal decomposition (**a**), coprecipitation (**b**), and commercially (**c**) are shown.

Three materials were used to assess the arsenic removal performance of nanoparticles, which are shown in Figure 1. TEM images demonstrate the general morphology and agglomeration patterns of each type of iron-oxide nanoparticles. IONPs produced by thermal decomposition (Figure 1a) were well dispersed and around 20 nm. IONPs prepared by coprecipitation (Figure 1b) showed small aggregates (around 200 nm) of nanoparticles (around 10 nm), even after being sonicated in water in preparation for the TEM grid. The commercially available IONPs (Figure 1c) showed large aggregates of IONPs, around 100 nm. The surface area was also measured to corroborate the size and agglomeration degrees observed with TEM. As expected, surface areas for commercial IONPs were much lower (4 m^2^/g) than those of IONPs prepared by coprecipitation (23 m^2^/g). Due to the limited amount of material, the surface area was not measured for IONPs prepared by thermal decomposition.

**Figure 2:**
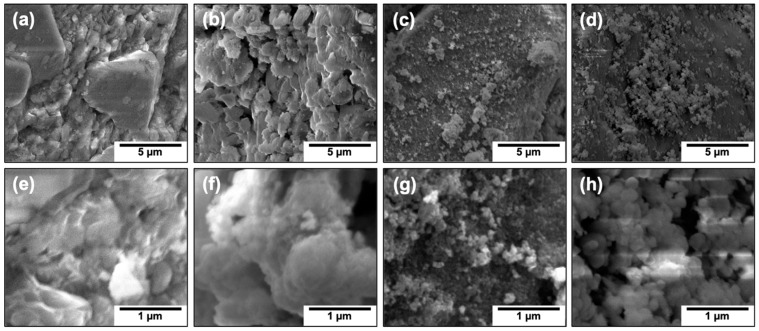
**SEM images of sand covered with IONPs of different agglomeration degrees:** SEM images of sand covered without and with IONPs at lower (**a**–**d**) and higher (**e**–**h**) resolution are shown. The sand surface (**a**,**e**) was covered with IONPs prepared via thermal decomposition (**b**,**f**), coprecipitation (**c**,**g**), or commercially (**d**,**h**).

The coprecipitation and commercial IONPs were mixed with sand to form active material, with nanomaterials comprising 20 wt%. IONPs prepared via thermal decomposition were added to form a 1 wt% mixture with sand. A lower concentration of IONPs was due to the difficulty in generating large amounts of materials with this synthetic method. The morphology of sand is presented in two magnifications in Figure 2a,e, for a point of reference. The homogeneity of the thermal decomposition IONPs in the sand mixture is displayed in Figure 2b, and areas highly populated with IONPs can be observed in Figure 2f. In Figure 2c, small and large aggregates of coprecipitation nanoparticles are observed; these nanoparticles can be spotted at higher magnification in Figure 2g. In Figure 2d, large aggregates of the commercial nanoparticles can be observed; the compactness of these aggregates is clear in Figure 2h. These SEM images show that the three columns can be utilized to effectively compare different degrees of agglomeration: large, compacted agglomerates are observed in commercial IONPs, a combination of large and small agglomerates are observed with IONPs prepared via coprecipitation, and IONPs prepared via thermal decomposition are highly dispersed and are, thus, barely visible on the sand.

The columns were loaded with corresponding mixtures and flushed with water before being exposed to the arsenic solution. Thermal decomposition IONPs moved through the column during flushing. Leaching of IONPs was observed during this process. Evidence of this movement can be clearly observed in Figure S5i. IONPs were transferred to the top of the column because of the upward flow. To minimize the leaching of nanoparticles during the experiment, the columns were run with a downstream flow; no IONPs were observed in the bottom of the column at the end of the experiment. To verify this, SEM samples of the three areas were taken. Figure S5ii shows the top part, where some aggregates of particles can be observed. Figure S5iii shows that the active bed is still very homogenous, and the roughness provided by the nanoparticles is kept after the experiment. Conversely, the bottom part shown in Figure S5iv is very smooth. Similar behavior, albeit to a smaller degree, was observed in the columns of IONPs prepared via coprecipitation and commercial IONPs. The column experiments were started only after the leaching of IONPs was no longer observed.

**Figure 3:**
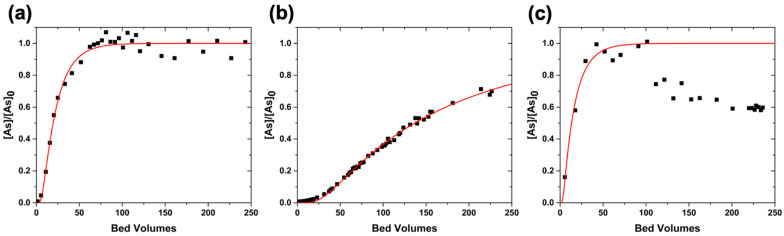
**Column experiments of sand covered with IONPs:** Small scale columns were performed with IONPs loaded in sand. The active beds were prepared by adding 1 wt% IONPs prepared via thermal decomposition (**a**), and 20 wt% IONPs prepared via coprecipitation (**b**) and commercially (**c**). A 100 ppb As solution (pH 7) was used as the feeding solution. The experimental data is shown with black squares while the red line represents the fitting model.

The performance of these columns is shown in Figure 3. For comparison, a column experiment with only sand is shown in Figure S6. The experimental data and the corresponding fitting are shown in Figure 3a for thermal decomposition nanoparticles, Figure 3b for the nanoparticles prepared by coprecipitation, and Figure 3c for the commercial nanoparticles. The details of the experimental parameters such as the length of the column (L), the size of the bed volume (BV), and the flow rate (Q) used are given in Table 1. The experimental data were fitted with a one-dimensional advection–dispersion model and solved with a simplification of the Ogata and Banks solution [27], as shown in Equation (1). The derived parameters, R (retardation factor) and D (coefficient of hydrodynamic dispersion), as well as the coefficient of determination (r) of the fitting model (red line), are also shown in Table 1.

(1) $$\frac{{\left[As\right]}_{x}}{{\left[As\right]}_{0}}=\frac{1}{2}erfc\left(\frac{RL-Q_{x}\left(EBCT\cdot x\right)}{\sqrt{4RD\left(EBCT\cdot x\right)}}\right)$$

In Equation (1), [*As*]*_x_/*[*As*]_0_ is the ratio of outlet concentration over initial arsenic concentration, which corresponds to the y-axis, and is dimensionless; *erfc()* is the complementary error function; *R* is the retardation factor, and is dimensionless; *L* is the length of the active area in the column, in cm; *Q_x_* is the linear velocity of the fluid through the column, in cm/min; *D* is the dispersion coefficient, in cm^2^/min; and instead of time, t, EBCT*x is used. *EBCT* is the empty bed contact time, in min/BV, and *x* is the number of bed volumes, BV, and corresponds to the x-axis in the graphs. All column experiments were performed at an *EBCT* of 2 min/BV.

As expected, IONPs with more aggregated particles, i.e., the commercial IONPs, demonstrated the worst performance (Figure 3c). For this fitting, only the points below 100 BVs were considered because of the decrease in the [As]/[As]_0_ ratio; this effect is not unusual in sand columns. The retardation factor (R) for this column was 13, and the hydrodynamic dispersion coefficient (D) was 73.3 cm^2^/min. The column with IONPs prepared via thermal decomposition showed a similar performance, with an R of 19 and a D of 6.3 cm^2^/min. The observed performance was even greater for the column of IONPs prepared with coprecipitation (Figure 3b). This column had an R of 145 and a D of 31.8 cm^2^/min. Considering that the column using IONPs prepared by means of thermal decomposition was loaded with a lower percentage of nanomaterials, and the leaching of IONPs was much higher than in the other two columns, two trends are apparent: when the particles are less aggregated, both the arsenic removal efficiency (observed with a larger retardation factor, R) and the homogeneity of the treatment (inversely proportional to the diffusion coefficient, D) improve. However, the deposition of individual nanoparticles in column systems results in the leaching of those particles during treatment, as observed during column packing (Figure S5).

### 3.2. Usage of Clusters of IONPs in Column Experiments

**Figure 4:**
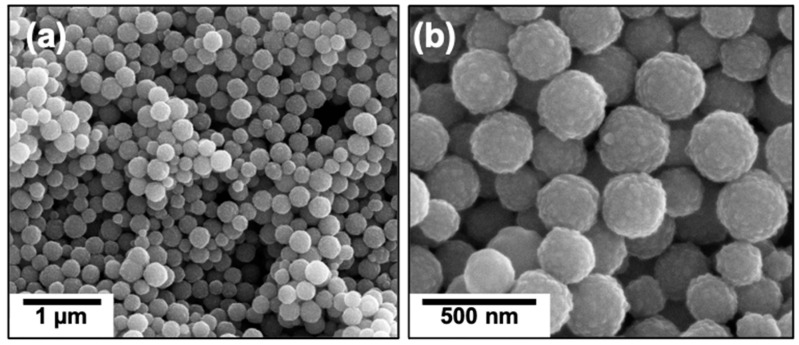
**Clusters of IONPs prepared via solvothermal synthesis:** SEM images of clusters of iron oxide nanoparticles at two different magnifications (**a**,**b**) are shown.

To achieve the right balance between large exposed surface area and agglomerate size that avoids the transport of the particles to the sand during water treatment, we proposed the use of clusters of iron oxide nanoparticles (cIONPs). Figure 4 shows SEM images of cIONPs prepared via solvothermal synthesis with diameters of 260 ± 40 nm. These particles demonstrate a hierarchical and controlled aggregation of nanoparticles [28], which led to an increase in surface area available for arsenic adsorption. The measured surface area, 44 m^2^/g, was higher than the surface area of IONPs (4 and 23 m^2^/g for commercial and synthesized through coprecipitation, respectively). Figure S7 shows low-resolution SEM images of the clusters homogeneously deposited onto the sand surface at different magnifications.

**Figure 5:**
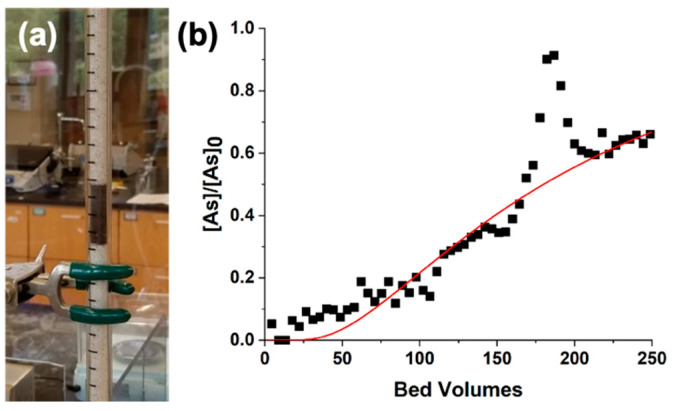
**Column experiment for sand covered with lusters of iron oxide nanoparticles**: image of the column with a 20%wt loading of clusters of iron oxide nanoparticles onto sand (**a**) used in a small scale column experiment (**b**). The experimental data is shown with black squares while the red line represents the fitting model.

Figure 5a shows the active bed with 20 wt% cIONPs on sand loaded in the column. In this case, the leaching of nanomaterial observed while flushing the column with water was minimal, comparable to the leaching observed with the commercial IONPs. Figure 5b shows the performance of cIONPs. The details of the experimental and derived parameters from the fitting are also provided in Table 1. This column showed better performance than the previous materials, with an R of 180 and a D of 1.8 cm^2^/min. For this fitting, the points between 170 and 190 BVs were ignored because of a sudden peak in the [As]/[As]_0_ ratio; the rapid increase in the arsenic concentration in the outlet resulted from stopping the column overnight. Due to this pause and the small bed volume, the mass transfer zone is distorted [29], and the outlet concentration starts to vary. For this reason, although the material was not saturated yet, the experiment was stopped. It is expected that a column with a larger bed volume and a higher nanomaterial loading will result in higher performance.

### 3.3. Comparison of Arsenic Adsorption on a Batch Setup

**Figure 6:**
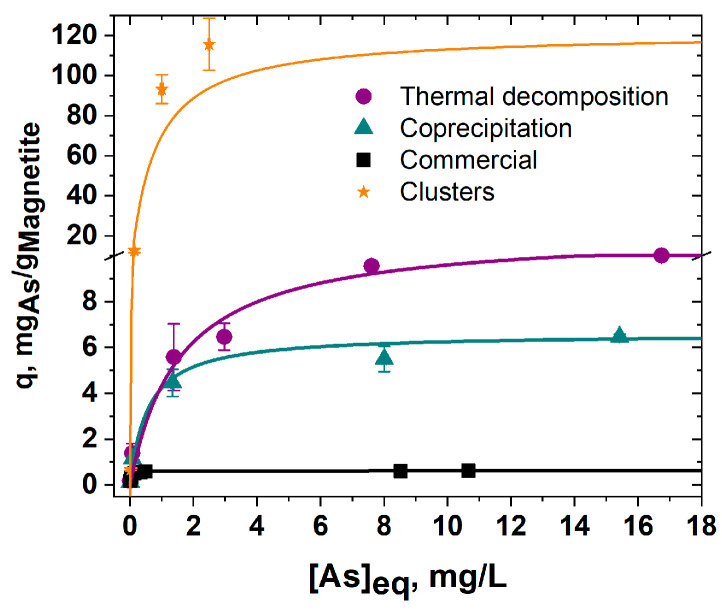
**Adsorption isotherms of IONPs prepared via different methods of synthesis:** adsorption isotherms are shown for IONPs prepared via thermal decomposition (purple circles), coprecipitation (cyan triangles), commercial (black squares), and solvothermal (orange stars).

To assess the adsorption capacity of each material, the arsenic removal efficiency was also measured in a batch experiment. Figure 6 shows the adsorption isotherm for each material. When comparing the effectiveness of IONPs, it is evident that the particles prepared in the lab had better performance. The difference in performance cannot be attributed only to size. The smallest IONPs prepared via coprecipitation were expected to perform the most efficiently; however, they were surpassed by the mid-sized IONPs produced by thermal decomposition. The large, commercial IONPs were the least effective. That said, there is no general trend that correlates NP size and arsenic removal efficiency. This trend disruption is believed to be a result of cohesive agglomeration patterns, as seen in Figure 1b. Thus, the effectiveness of arsenic removal is determined by both the size and the aggregation state of IONPs. This is strongly supported by the performance of the clusters of nanoparticles, whose adsorption capacities at similar concentrations are more than 10 times higher.

The experimental data were fitted to the Langmuir model (Equation (2)), which provides the parameters q_max_ and k_L_. Table 2 summarizes the results. q_max_ corresponds to the maximum capacity of the analyte that the material can adsorb, and k_L_ is a constant related to the system. q_10_ and q_100_ are also reported and represent the adsorption capacities predicted by the fitting at concentrations of [As] = 10 μg/L and 100 μg/L. The significance of these values is that q_100_ is the maximum expected capacity for a column that is fully saturated when in contact with the arsenic concentration used for the column experiments, whereas q_10_ would be the expected capacity for a column that meets the Environmental Protection Agency (EPA) maximum contaminant level (MCL) [30].

(2) $$q=q_{\max}\frac{C_{\mathrm{eq}}k_{L}}{1+C_{\mathrm{eq}}k_{L}}$$

For all of the studied nanoparticles, the adsorption capacities when arsenic was present at 10 ppb (q_10_) were not different (0.1 mg/g). However, when the arsenic concentration increased to 100 ppb, the capacities (q_100_) increased with decreasing nanomaterial size: coprecipitation IONPs had the highest q_100_, 1.00 mg/g, followed by IONPs prepared via thermal decomposition, with 0.7 mg/g, and the commercial nanoparticles, with 0.5 mg/g. On the other hand, the degree of agglomeration had the largest impact on the maximum adsorption capacity (q_max_), as the highest q_max_ was observed in the fully dispersed IONPs prepared via thermal decomposition, at 11.1 mg/g, followed by 6.6 mg/g for coprecipitation nanoparticles and a very low 0.6 mg/g for the aggregated commercial nanoparticles. These adsorption capacities are in the same order as that reported in other studies for iron oxide nanoparticles [4].

More importantly, all of the adsorption capacities (q_10,_ q_100,_ and q_max_) were more than 10 times larger for cIONPs. The maximum adsorption capacities observed for cIONPs were similar to the highest adsorption capacities reported for other iron oxide nanomaterials, which corresponds to a Zr-doped β-FeOOH nanomaterial [31].

## 4. Conclusions

In this study, we analyzed the effect of using IONPs with different grades of agglomeration on their arsenic removal behavior in filter-alike setups when deposited on a sand support medium. Thermal decomposition was used to generate well dispersed IONPs, while coprecipitation generated smaller nanoparticles that quickly form small aggregates. We compared the freshly synthesized IONPs to commercially available IONPs, which tend to be larger and highly agglomerated. The active materials, i.e., sand covered with IONPs, were then used in continuous flow column experiments. As expected, smaller nanoparticles had higher capacities, as there was more surface area available for adsorption. However, their capacities were diminished when agglomeration was present. Furthermore, having well-dispersed nanoparticles over a support media resulted in IONPs leaching from the column, which might affect water treatment in the long term. For this reason, we proposed the use of clusters of nanoparticles, which can be synthesized with a solvothermal method. In cIONPs, aggregation of small nanoparticles is controlled, which maximizes the exposed surface area for arsenic adsorption. An improved performance was observed in the column experiment while also minimizing the leaching of nanomaterials into the treated water. This enhancement was also observed in the arsenic adsorption capacities obtained from a batch experiment, the results of which showed that the maximum arsenic adsorption capacity for the cIONPs was 121.4 mg/g, while the capacities for IONPs prepared via thermal decomposition and coprecipitation were 11.1 and 6.6 mg/g, respectively.

Our results showed that the use of clusters of nanoparticles allows the exploitation of controlled agglomeration to maximize the high surface areas, making IONPs very promising for environmental applications while minimizing the implementation challenges that prevent the use of nanoparticles in filter setups. This research revealed that clusters of IONPs can be used to effectively remove arsenic in column experiments. The use of clusters of nanoparticles with other compositions opens up the possibilities for multiple water remediation applications. This study is the first step to bringing nanomaterials to real-world environmental applications.

## Figures and Tables

**Table 1:** **Summary of the parameters for column experiments.**

	L (cm)	BV (mL)	Q (mL/min)	R	D (cm^2^/min)	r^2^
Thermal decomposition	7	5.5	2.8	19	6.3	0.976
Coprecipitation	10.5	8.3	4.1	145	31.8	0.990
Commercial	25.5	20.0	6.4	13	73.3	0.961
Clusters of Nanoparticles	3.5	2.8	1.4	180	1.8	0.856

A summary of the parameters used (L, BV, and Q) and obtained (R, D, and r^2^) by fitting the experimental data from Figure 3 to an advection–diffusion model is shown. The experimental parameters include length of the column (L), the size of the bed volume (BV) size, and the flow rate (Q), while R (retardation factor) and D (coefficient of hydrodynamic dispersion), as well as the coefficient of determination (r), are derived from the fitting model.

**Table 2:** **Summary of the parameters of isotherm experiments for IONPs.**

	q_max_ (mg/g)	k_L_ (L/mg)	q_10_ (mg/g)	q_100_ (mg/g)
Thermal decomposition	11.1	0.6	0.1	0.7
Coprecipitation	6.6	1.8	0.1	1.0
Commercial	0.6	31.4	0.1	0.5
Clusters of Nanoparticles	121.4	1.4	1.6	14.6

A summary of the parameters (q_max_ and k_L_) obtained by fitting the experimental data to a Langmuir model (Figure 6) is shown. The adsorption capacities observed at 10 ppb and 100 ppb, q_10_ and q_100_, respectively, are also shown.

## Data Availability

Data are contained within the article or Supplementary Materials.

## Supplementary Materials

The following supporting information can be downloaded at: https://www.mdpi.com/article/10.3390/nano12091598/s1. Details on the synthesis and characterization of the nanomaterials used in this study. Figure S1: Assembly of the columns, Figure S2: Sand covered with IONPs prepared via thermal decomposition after treatment with super hydride, Figure S3: Experimental procedure for adsorption isotherms, Figure S4: Modification of adsorption isotherms using IONP solutions. Figure S5: Column using sand covered with IONPs prepared via thermal decomposition, Figure S6: Column experiment for pristine sand, Figure S7: SEM images of active bed of sand covered with cIONPs [32,33,34].
