# Supplementary material for: The Effect of Agglomeration on Arsenic Adsorption Using Iron Oxide Nanoparticles
Source: Nanomaterials (Basel). 2022 May 9;12(9):1598. doi: 10.3390/nano12091598 (PMC9105002; doi:10.3390/nano12091598)
Supplement: Supplementary file 1 [file nanomaterials-12-01598-s001.zip › nanomaterials-1685821-supplementary.pdf]

Supporting information (SI)

for the article entitled:

The Effect of Agglomeration on Arsenic Adsorption Using Iron Oxide Nanoparticles

William R. Diephuis, Anna L. Molloy, Lindsey L. Boltz, Tristan B. Porter, Anthony Aragon Orozco, Reina Duron, Destiny Crespo, Luke J. George, Andrew D. Reiffer, Gabriela Escalera, Arash Bohloul, Carolina Avendano, Vicki L. Colvin, Natalia I. Gonzalez-Pech

1. Materials.

1.1. Synthesis of nanomaterials. Iron(III) chloride ($FeCl_3$, 97%), iron(II) chloride tetrahydrate ($FeCl_2•4H_2O$, 98%), iron(III) chloride hexahydrate ($FeCl_3•6H_2O$, 97%), oleic acid (OA, technical grade, 90%), 1-octadecene (ODE, technical grade, 90%), urea, and ethylene glycol (anhydrous, 99.8%) were purchased from Sigma-Aldrich; sodium oleate was acquired from Fisher Scientific. Ethanol 190 proof (95%, KOPTEC) was acquired from VWR. Hexanes (certified ACS grade) and acetone (certified ACS grade) were purchased from Fisher Scientific. Super hydride solution (1.0M lithium triethylborohydride in THF) was procured from Sigma-Aldrich.

1.2. Commercial arsenic sorbents. Ferroxide Black 78P was purchased from Rockwood Pigments.

1.3. ICP analysis. Nitric acid (HNO_3, 70%, purified by redistillation, >99.999% trace metals basis) and hydrogen peroxide (H_2O_2, 30wt% in H_2O) were required for sample digestions. Standards for ICP were purchased in Sigma-Aldrich, including arsenic (TraceCERT, 1000 mg/L As in nitric acid), iron (TraceCERT, 1000 mg/L Fe in nitric acid), sulfur (TraceCERT, 1000 mg/L S in H_2O), silver (TraceCERT, 1000 mg/L Ag in nitric acid), phosphorous (TraceCERT, 1000 mg/L P in H_2O), chromium (TraceCERT, 10,000 mg/L Cr in nitric acid), lead (TraceCERT, 1000 mg/L Pb in nitric acid), mercury (TraceCERT, 1000 mg/L in nitric acid), silicon (TraceCERT, 1000 mg/L Cd in nitric acid), multielement standard solution 4 for ICP (TraceCERT, 40 mg/L Al, 40 mg/L As, 100 mg/L B, 40 mg/L Ba, 10 mg/L Be, 10 mg/L Cd, 10 mg/L Co, 20 mg/L Cr, 20 mg/L Cu, 100 mg/L Fe, 10 mg/L Mn, 20 mg/L Ni, 40 mg/L Pb, 100 mg/L Se, 100 mg/L Tl, 40 mg/L V, and 100 mg/L Zn in 10% nitric acid), and multielement standard solution 3 for ICP (TraceCERT, 2000 mg/L Ca, 200 mg/L K, 400 mg/L Mg, and 1000 mg/L Na in 5% nitric acid).

2. Synthesis of nanoparticles

2.1. Thermal decomposition. The methodology utilized to produce nanoparticles via thermal decomposition is based on the procedure previously described by the Colvin [32] and Hyeon [33] groups. This synthesis technique consists of two reaction steps: preparation of an iron oleate precursor and the synthesis of nanoparticles. Iron oleate was first prepared by dispersing a mixture of ferric chloride hexahydrate ($FeCl_3•6H_2O$, 30mmol, 8.1g) and sodium oleate (120mmol, 36.5g) in 75 mL of DI water. Afterward, 75 mL of ethanol was added and stirred for 10 minutes. After the addition, the polar phase was clear, and the organic phase

was dark orange. Lastly, 150 mL of hexane was added. The reaction was refluxed at 60°C for 4 hours. The dark orange solution was washed five times with 30 mL of DI water. Once clean, the iron oleate solution was divided into 10 mL portions and collected in 50 mL centrifuge tubes. Acetone (30 mL) was added to each of the tubes and mixed together. The tubes were then centrifuged at 11,000 rpm for 15min to isolate the iron oleate.

Next, the prepared iron oleate precursor was utilized for thermal decomposition synthesis of low-dispersion magnetite nanoparticles (nMag). Briefly, 1 mmol of the iron oleate precursor was mixed with 4 mmol of oleic acid and 10 mL of octadecene (ODE). The reaction mixture was heated at 315°C for 2 h to produce monodispersed iron oxide nanocrystals. The iron oxide nanocrystals were precipitated from ODE using acetone and then redispersed in hexanes.

2.2. *Co-precipitation.* A 0.2 M Fe^{+} and 0.1M Fe^{+} was prepared by mixing $FeCl_3$•$6H_2O$ (2.7g) and $FeCl_2$•$4H_2O$ (1.0g) with 50 mL DI water. The solution was deoxygenated, heated at 80°C, and stirred for 30 minutes. This solution was drawn into a 60 mL syringe and added dropwise to 200 mL of 1M NaOH. The reaction mixture was heated at 80°C and stirred at 800 rpm during Fe^{+} addition. Following the addition, the solution was kept at 80°C and stirred for 30 additional minutes. After 30 minutes, the solution was removed from heat and stirred (400 rpm) until it reached room temperature. The reaction mixture was transferred to a 50 mL centrifuge tube with a transfer pipette. A magnet was utilized to concentrate the nanoparticles. The particles were redispersed in 100 mL of DI water and vigorously mixed by hand. The particles were then collected again with the magnet. This cleaning process was repeated until the pH of the supernatant was 7. Once clean, the particles were dried and stored. Particles remained stable and usable for about one to two weeks.

2.3. *Solvothermal synthesis*. The methods utilized in this synthesis technique were adapted from work previously reported by Yu in 2006 [34]. A Fe^{+} (100 mM) solution was prepared by combining $FeCl_3$•$6H_2O$ (2.29g) with 85 mL ethylene glycol. The solution was stirred at 400 rpm and heated to 65°C to speed up the iron chloride dissolution rate. After stirring for 30 minutes, 2 mM PAA-NA (1.02g) was added to the solution. The stirring was increased to 800 rpm for 45 minutes. 1M of urea (5.10 g) was added last to the solution, which was left to stir for 45 minutes. Teflon liners were loaded with 20 mL of the reaction mixture. The Teflon liner was placed in a stainless steel autoclave. The autoclave was then heated in an oven for

24 hours. After 24 hours, the autoclave was removed from the oven and allowed to cool to room temperature. The reaction mixture was transferred to a 20 mL vial with a transfer pipette. A magnet was utilized to collect the cluster and dispose of the supernatant. The clusters were then dried and stored.

2.4 Coating removal. Nanoparticles prepared via thermal decomposition have a surface coating of oleate chains. The following process was used to remove the oleate from the NP surfaces. For this procedure, 5 mL NP solutions prepared via thermal decomposition in hexanes were placed in 50 mL centrifuge tubes. Then, 5 mL of super hydride solution was taken with a 12 mL syringe (water and humidity should be avoided for safety reasons); then, the needle was carefully replaced with a 0.2 μm PES syringe filter, and the super hydride solution was added slowly to the NPs prepared via thermal decomposition. Bubbles were observed during addition. The tube was closed and placed in a bath sonicator for 10 min. Next, 20 mL of ethanol was slowly added to the tube, and bubbling was observed. The mixture was sonicated for 10 more minutes. Acetone was used to bring the volume to 45 mL, and the tube was centrifuged at 11,000 rpm for 15min. Then, 5 mL hexanes were added and mixed with the help of a vortex. This procedure was repeated at least 3 times until bubbles were not observed during super hydride addition.

To remove surface coatings from solvothermal nanomaterials, a similar procedure was used. Between 0.9 and 1.5g of dry nanomaterials was added to a 50 mL centrifuge tube. Using a 12 mL syringe, super hydride solution was added to the nanomaterials, and bubbling was observed. Super hydride was added gradually to the tube until no further bubbling was observed. To neutralize excess super hydride, ethanol was slowly added to the tube until no further bubbling was observed. The resulting mixture was centrifuged at 4,000 rpm for 10 min, the liquid was drained, and the nanomaterials were dried under a vacuum. This procedure was also used to remove the surface coating from mixtures of sand and NPs.

3. Characterization

3.1. Transmission electron microscopy. TEM samples were prepared by dropping the solution (10 to 20 μL) on ultra-thin carbon type-A 400 mesh copper grids (Ted Pella Inc.), allowing them to dry slowly. TEM micrographs were taken on a JEOL 2100 field-emission gun TEM operated at 200 kV with a single tilt holder. Typically, the size and size distribution data were

obtained by counting more than 1000 particles using ImageJ, in images taken at 40K magnifications.

3.2. Brunauer–Emmett–Teller (BET) surface area analysis. BET surface area analysis was carried out on an automated Quantachrome Autosorb 3B Surface Analyzer. A specific mass of solid (a few milligrams) was introduced to a glass bulb-ended tube and heated under vacuum at 200 °C for several hours to remove surface adsorbed contaminants. Then, it was cooled with liquid nitrogen and analyzed by measuring the volume of N2 gas adsorbed at specific pressures. The surface area was calculated automatically using an Autosorb 3 software program.

3.3. Scanning electron microscopy. Solids were placed on SEM mounts using carbon tape without gold surface coating. SEM images were either obtained on an FEI Quanta 400F Field Emission Scope at 10.0 kV or a Magellan 400 XHR SEM at 15.0 kV. Images at magnifications between 100x and 100,000x were usually acquired.

Low-magnification SEM images were obtained using a Hitachi TM3000 microscope at 15kV. Images were taken at magnifications of 100x, 1,000x, and 5,000x.

3.4. Inductively coupled plasma–optical emission spectroscopy. The particle concentrations were measured by a PerkinElmer Optima 8300 Inductively Coupled–Plasma Optical Emission Spectrometer (ICP–OES), equipped with an autosampler or a PerkinElmer Avio 200 Inductively Coupled Plasma (ICP) Optical Emission Spectrometer. All measurements were carried out with calibrations with an R>0.9999 and a LOQ lower than 0.2 mg/L. Fe was measured simultaneously in two wavelengths, 273.955 nm and 238.204 nm, 238.204 nm, in a radial mode. Calibration curves usually had standards between 1 and 60 mg/L.

Sample preparation was carried out by adding 1 mL of HNO_3 trace metals to 50 µL of in-hexanes thermal decomposition NP solutions. The sample kept mixing for 1h under mild heating (around 70°C, using a hot plate); when needed, 1 mL extra of nitric acid was added and mixed for one more hour. When only one clear aqueous phase was left, the solution was transferred to a 10 mL volumetric flask where it was diluted with DI water to the flask's mark. Then, samples were filtered with a 0.2 µm PES syringe while being added to a 15 mL centrifuge tube.

High arsenic concentrations (ppm level) were measured also by ICP–OES. All measurements were carried out with calibrations with an R> 0.999, and a LOQ lower than 0.1 mg/L for As. Calibration curves usually had standards between 0.5-20 mg/L for As.

3.5. Inductively Coupled Plasma–Mass Spectrometry. Arsenic and other element concentrations were measured by a PerkinElmer Nexion 300 Inductively Coupled Plasma–Mass Spectrometer (ICP–MS) equipped with an autosampler. All measurements were carried out with calibrations with an R> 0.9999 and a LOQ lower than 0.2 µg/L. Arsenic was measured both by itself or in a multielement solution. In both cases, calibration curves usually had standards between 1 and 200 µg/L of As. Sample preparation consisted of the acidification of the solutions: briefly, 0.15 mL of 70% HNO_3 (trace metals basis) were added to 10 mL of the solution to obtain a 1% nitric acid solution. All samples were filtered prior to acidification with a 0.2 µm PES syringe filter.

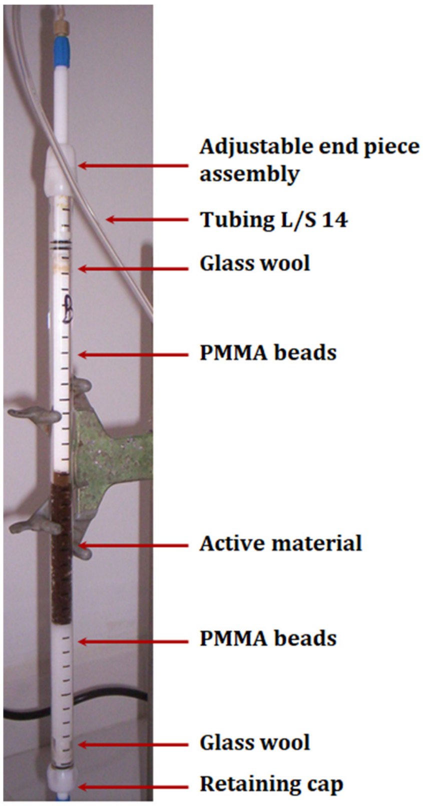

Figure S1. Assembly of the columns

The column was armed from bottom to top. A retaining cap was well adjusted. First, 1 cm^3 of glass wool was packed. Then, at least 10 cm^3 of support material (PMMA beads or sand) was added. The desired volume of active material was then added and packed compactly. A second layer of support material was added with another centimeter of glass wool. An adjustable end piece assembly was used at the top, to adjust any change in compactness observed when water starts to flow.

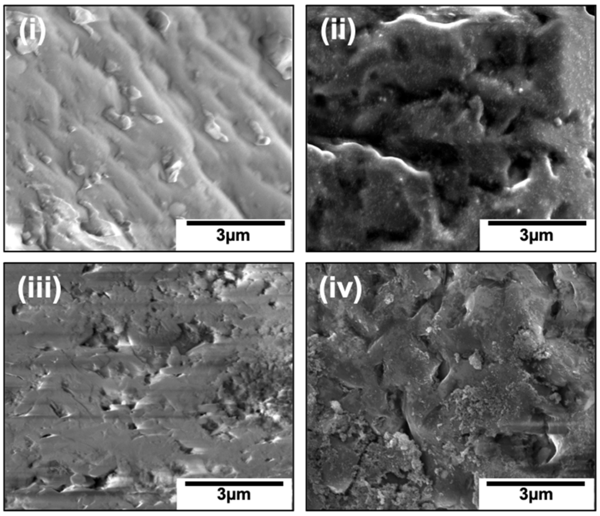

Figure S2. Sand covered with IONPs prepared via thermal decomposition after treatment with super hydride.

High-magnification SEM images of sand (i, iii) and thermal decomposition NP-covered sand (ii, iv) before and after an external treatment with super hydride solution are shown.

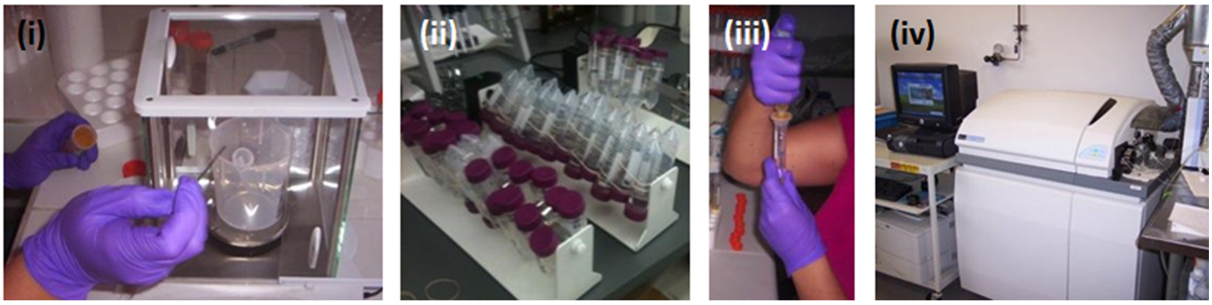

Figure S3. Experimental procedure for adsorption isotherms.

The solid was weighed into the centrifuge tube (i) where the solution of interest would be added. Then, the tube was mechanically mixed for 4 h (ii). When finished, the solution was filtered using a syringe filter and transferred to a new 15 mL centrifuge tube (iii). After acidification, the sample concentration was measured via ICP.

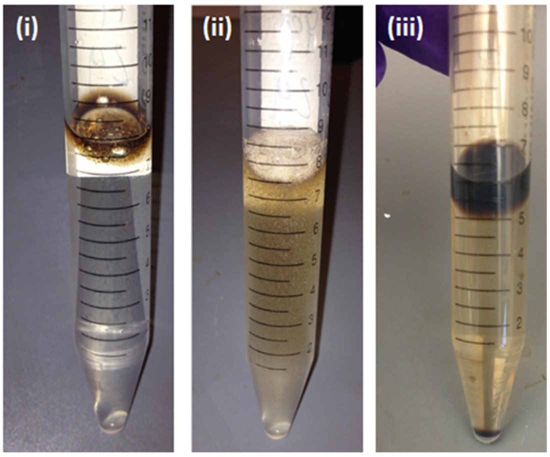

Figure S4. Modification of adsorption isotherms using IONP solutions.

(i) The NP solution was added to the arsenic solution. (ii) During mixing, the sample seemed homogeneous. (iii) After the mixing was stopped, the remaining hexane solution could be separated from the aqueous phase. The presence of precipitate indicates that some arsenates replaced carboxylate groups.

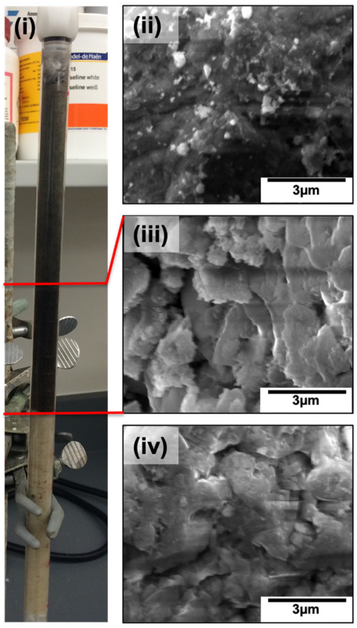

Figure S5. Column using sand covered with IONPs prepared via thermal decomposition.

Image of a 1 %wt nanomaterial–sand column after experiment (i) is shown. The SEM images of the three areas are shown: top (ii), active bed (iii), and bottom (iv).

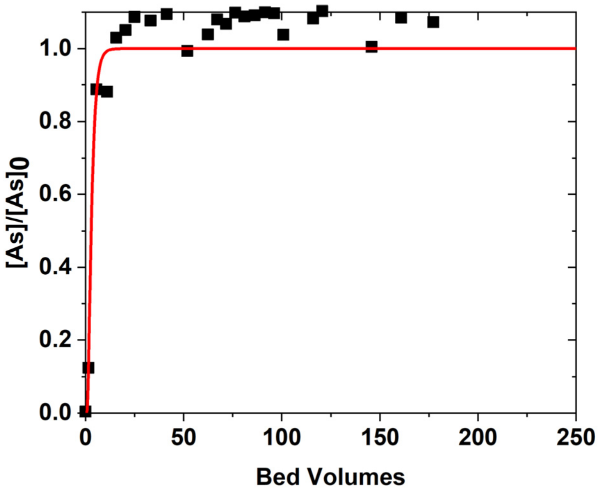

Figure S6. Column experiment for pristine sand.
A small-scale column was performed with only sand. A 100 ppb As solution (pH 7) was used as the feeding solution.

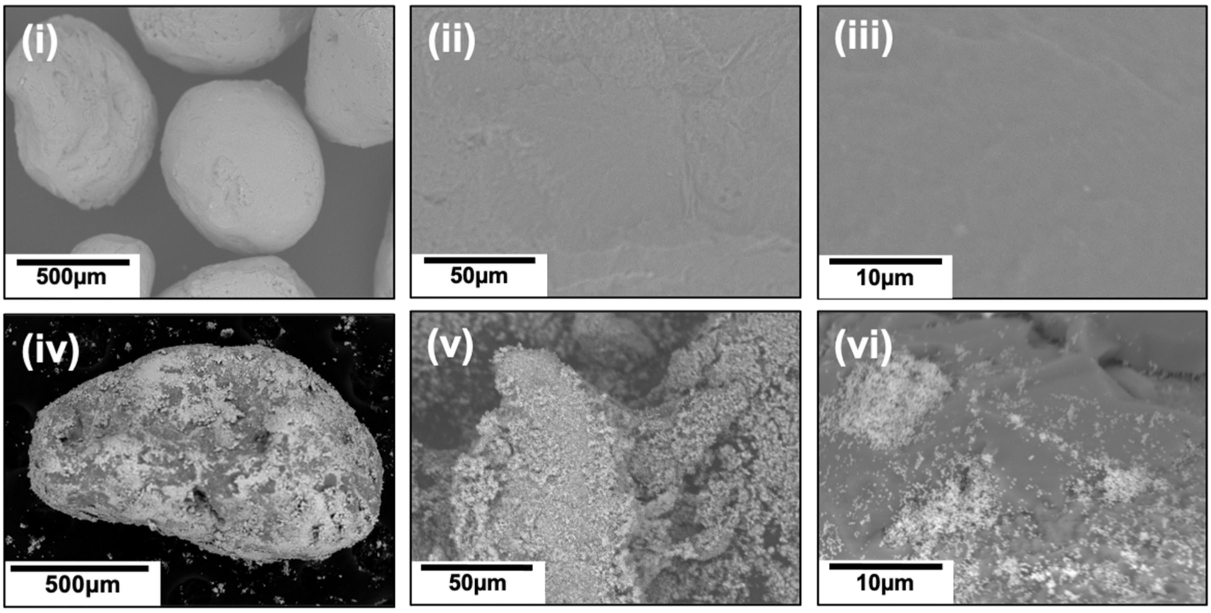

Figure S7. SEM images of active bed of sand covered with cIONPs.
SEM images of pristine (i-iii) and cluster-covered (iv-vi) sand are shown. The material is shown at multiple magnifications to compare the deposition of the clusters.

References:

[32] Yu, W. W.; Falkner, J. C.; Yavuz, C. T.; Colvin, V. L. Synthesis of Monodisperse Iron Oxide Nanocrystals by Thermal Decomposition of Iron Carboxylate Salts. *Chem. Commun.* **2004**, No. 20, 2306–2307. https://doi.org/10.1039/B409601K.

[33] Park, J.; An, K.; Hwang, Y.; Park, J.-G.; Noh, H.-J.; Kim, J.-Y.; Park, J.-H.; Hwang, N.-M.; Hyeon, T. Ultra-Large-Scale Syntheses of Monodisperse Nanocrystals. *Nat. Mater.* **2004**, *3* (12), 891–895. https://doi.org/10.1038/nmat1251.

[34] Yu, D.; Sun, X.; Zou, J.; Wang, Z.; Wang, F.; Tang, K. Oriented Assembly of Fe3O4 Nanoparticles into Monodisperse Hollow Single-Crystal Microspheres. *J. Phys. Chem. B* **2006**, *110* (43), 21667–21671. https://doi.org/10.1021/jp0646933.
